# The synbiotic mixture of *Bacillus licheniformis* and *Saccharomyces cerevisiae* extract aggravates dextran sulfate sodium induced colitis in rats

**DOI:** 10.1186/s12917-022-03479-y

**Published:** 2022-11-16

**Authors:** Gamal A. Salem, Amany Abdel-Rahman Mohamed, Wael A. M. Ghonimi, H. M. Abdallah, Nasreddin R. Rhouma, Reem I. Ali

**Affiliations:** 1 Department of Pharmacology, Faculty of Veterinary Medicine, Zagazig University, P.O. Box 44519, Zagazig, Egypt; 2 Departments of Forensic Medicine and Toxicology and Faculty of Veterinary Medicine, Zagazig University, Zagazig, 44519 Egypt; 3 Department of Histology and Cytology, Faculty of Veterinary Medicine, Zagazig University, Zagazig, 44519 Egypt; 4 Department of Microbiology and Immunology, Faculty of Veterinary Medicine, Zagazig University, Zagazig, 44519 Egypt; 5 Department of Micobiology, Faculty of Science, Misurata University, Misurata, P.O. Box 2478, Libya; 6 Department of Microbiology and Immunology, Faculty of Veterinary Medicine, Banha University, Banha, 13518 Egypt

**Keywords:** Probiotic, Prebiotic, Ulcerative colitis, TNFα, INFγ, IL-1β

## Abstract

**Background:**

Uncertain effects of probiotics and/or prebiotics have been reported in experimental and clinical colitis. This study aims to examine the effects of a synbiotic combination comprising *Bacillus licheniformis* DSM 17236 and *Saccharomyces cerevisiae* cell wall extract on dextran sulfate sodium (DSS)-induced colitis in Sprague Dawley rats.

**Methods:**

Acute colitis was induced in rats by oral administration of DSS 3.5% for 7 days. Fifty rats were divided equally into five groups; one control group and the other groups were induced with colitis and treated with or without the tested synbiotic, mixed with diet, for 28 days and sulfasalazine (100 mg/kg) via intragastric tube once daily for 14 days.

**Results:**

Symptomatically, the synbiotic administration raised the disease activity index (DAI) to comparable scores of the DSS group, specially from the 2nd to 7th days post DSS intoxication. It also induced a significant (*p* < 0.05) amplification of WBCs, myeloperoxidase (MPO), malondialdehyde (MDA), nuclear factor kappa B (NF-kB) expression and proinflammatory cytokines tumor necrosis factor alpha (TNFα), interferon gamma (INFγ), and interleukin-1 beta (IL-1β) while depressed the antioxidant enzymes glutathione peroxidase (GPx), catalase (CAT), and superoxide dismutase (SOD) when compared with the DSS and control groups. The DSS intoxicated and Synbiotic+DSS groups showed desquamations of the covering epithelium, noticeable diffuse leukocytic infiltrations, sever catarrhal enteritis, ischemic colitis with diffuse coagulative necrosis of the entire colonic mucosa. Contrarily, sulfasalazine proved to be effective in the reduction of the tested inflammatory markers and the pathological degenerative changes of the DSS ulcerative colitis.

**Conclusion:**

The examined synbiotic did not ameliorate but aggravated the DSS-induced colitis, so it should be subjected to intensive experimental and clinical testing before their use in animals and human.

## Background

Inflammatory bowel disease (IBD) involves a group of multifactorial disorders including two major forms; Crohn’s disease (CD) and ulcerative colitis (UC), that affect the integrity of the intestinal mucosa [1, 2]. Although the pathogenesis of IBD is not clear, there is strong evidence that the breakdown of intestinal mucosal homeostasis is correlated with exaggerated inflammatory responses against intestinal lumen antigens in genetically sensitive individuals [2, 3].

Cytokines specially TNFα, INFγ and interleukin 1-beta (IL-1β) are key components of IBD pathogenesis by mediating the communication between innate and adaptive immune cells and stimulate the recruitment of inflammatory cells to the intestinal mucosa [4–7]. DSS destroys the intestinal epithelial lining mucosa and increases the exposure of the innate immune cells to intestinal microbiota leading to acute inflammatory responses, thus it is considered the most widely used rat model for human IBD [8, 9].

Recent approaches dedicated that oxidative stress (OS) including reactive oxygen and nitrogen species (ROS, RNS) play a deleterious role in the pathogenesis of IBD [10]. Gut comprising the microbiome, food stuffs and immune cell interactions constitutes a potential source for pro-oxidants [11]. Intestinal cells harbor a defense enzymatic system including SOD, GPx, and CAT that can scavenge the free radicals and prevent lipid peroxidation and further cell damage [12]. The excessive OS could deplete the antioxidant competencies in IBD patients even in asymptomatic stage of the disease [13]. It is worthy noted that OS could lead to immune system activation and inflammatory process that correlate to tissue injury and ulcerations in CD and UC [14].

The current medications of IBD involving immunosuppressants and anti-inflammatory drugs as sulfasalazine, corticosteroids and anti-TNFα (tumor necrosis factor alpha) antibody are used to depress the aberrant immune responses and inflammatory cascades [15]. However, the lack of efficacies and the adverse effects associated with these drugs over prolonged treatment imply for their limited use [16]. Recent studies suggested that modulation of the OS and immune response may be valuable in the treatment of IBD since inflammation with OS contribute to tissue damage [17].

Probiotics and prebiotics have been used more or less successfully in IBD patients and their protective and therapeutic effects mainly depend on the strains used [18–20], where each probiotic strain may have discrete immunoregulatory properties and can be classified into two groups according to their influence on the immune system: one exhibiting immunostimulating activities and the other anti-inflammatory properties [19]. Bacillus species have been used as probiotics in Italy since 1985 as an OTC medicinal supplement [21]. The most broadly examined strains are *Bacillus subtilis, Bacillus clausii, Bacillus cereus, Bacillus coagulans* and *Bacillus licheniformis*. Being a spore former, it has the advantages of heat stability and can be stored at room temperature, in addition to its ability to survive the acidity of the stomach [22, 23]. According to Sorokulova et al. *Bacillus licheniformis* were found to be safe in the in-vivo toxicity studies, non-toxinogenic, and sensitive to the antibiotics listed in european food safety authority (EFSA), therefore it may be considered as non-pathogenic and safe for human consumption [24].

Veterinary studies showed that *Bacillus licheniformis* ameliorated diarrhea in growing pigs [25], stimulated the immune system in challenged piglets with *E. coli* [26], increased milk production, milk fat, as well as protein percentage in lactating ewes [27], enhanced growth performance and normalized intestinal microbiota in necrotic enteritis of broilers [28], improved egg production and quality in laying hens [29], and diminished intestinal gas formation and foul fecal odor in dogs [30].

Prebiotics are nondigestible dietary substances that have the ability to stimulate growth of native and probiotic bacteria [31]. Potentially, the synbiotic combinations of probiotic and prebiotic were proved to have benefits more than that of the probiotic alone, because the prebiotic can improve the colonization, growth, or affectivity of the probiotic species [32, 33]. Prebiotics including inulin, fructooligosaccharide (FOS), and Yeast cell wall extract (Mannan oligosaccharides and glucans) have variable effects when tested in experimental and clinical IBD [32, 34–36].

As far as our research team knows, a scarcity of studies is available in scientific literature regarding the effect of *Bacillus licheniformis* on the chemically induced colitis. Therefore, the present study was conducted to investigate the ability of the synbiotic mixture of *Bacillus licheniformis* DSM 17236 and an extracted prebiotic from cell walls of the baker yeast, *Saccharomyces cerevisiae*, to modify DSS-induced experimental colitis in Spargue Dawley rats in comparison to the sulfasalazine drug. The assessment included the detection of DAI and the colonic tissue levels of MPO, oxidative stress parameters, NF-kB expression. Furthermore, the leukocytic count and serum inflammatory cytokines (TNFα, INFγ, and IL1β) level were assayed. Histopathological examination of intestinal colitis was also performed.

## Results

**Fig. 1:**
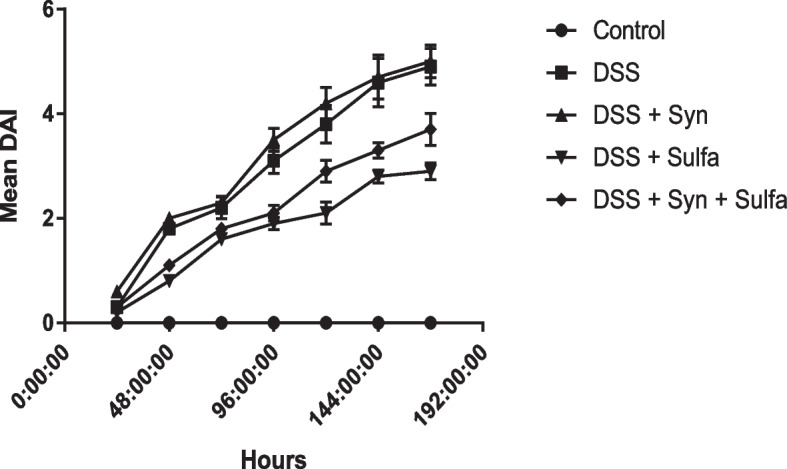
Disease activity index (DAI) of the 5 groups (Control group, DSS group, DSS + Syn group, DSS + Sulfa group, and DSS + Syn + Sulfa group) during the development of DSS-induced colitis between Days 1 and 7 from DSS administration. DAI score is expressed as the mean ± SEM (*n* = 10 treatments)

**Fig. 2:**
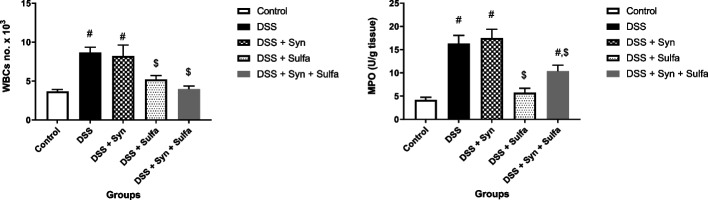
The effect of synbiotic (B.licheniformis and yeast extract) and or sulfasalazine (100 mg/kg) on WBCs count in blood and colonic tissue MPO level (U/g tissue) against DSS induced colitis in rats. Data are expressed as the mean ± SEM. # indicates significant difference (*P* < 0.05) compared to control group. $ indicates *P* < 0.05 compared to DSS group (*n* = 10 treatments)

The administration of 3.5% of DSS in drinking water for 7 days developed the symptoms of the inflammatory conditions in colon in the form of wasting, loss of weight, diarrhea, and rectal bleeding. In parallel with the previous symptoms, the DAI (Fig. 1) markedly increased with the 2nd day after DSS administration in comparison with the control untreated rats. While the animals from control group showed no weight loss, changes in stool consistency or presence of fecal occult blood during the 7 days of treatment. The DSS group induced a significant elevation (*P* < 0.05) of WBCs count (Fig. 2) in blood compared to the control group.

**Fig. 3:**
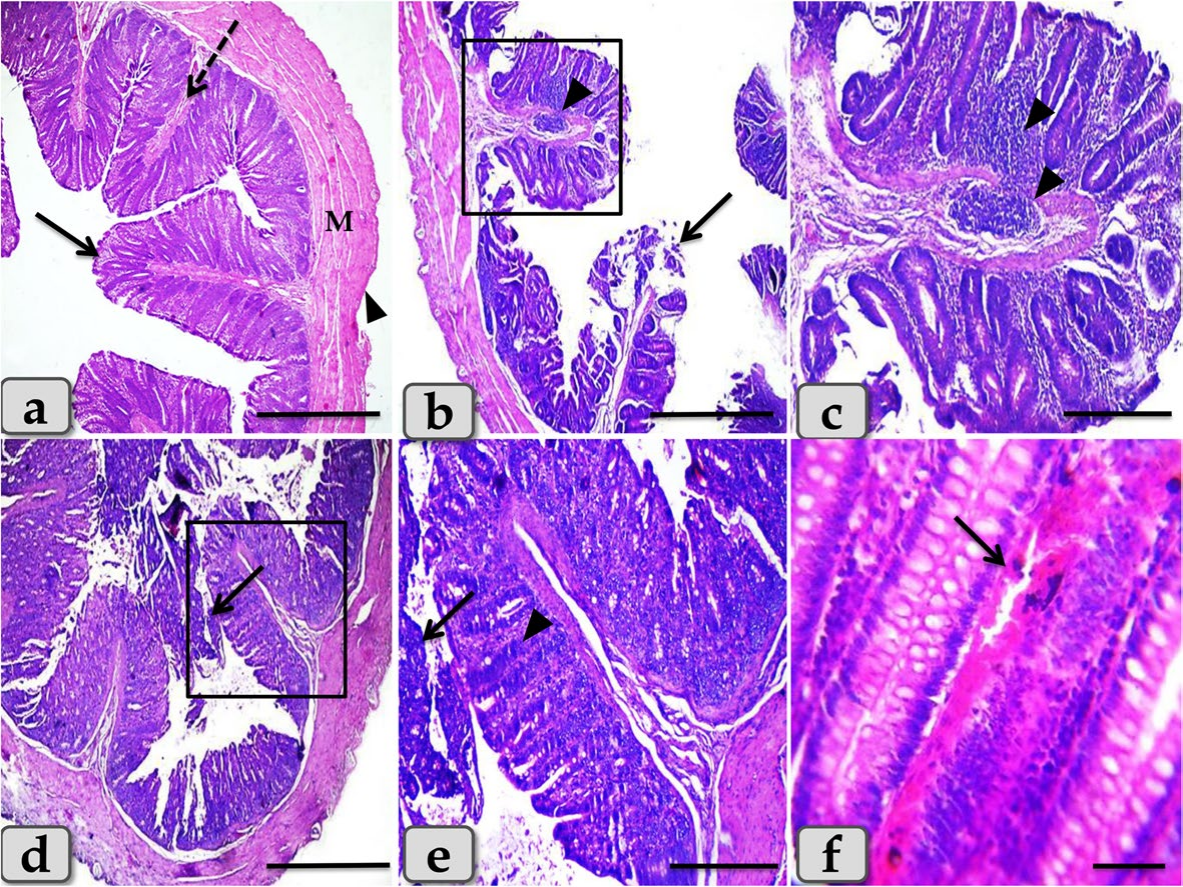
Illustrative photomicrograph of colon of the negative control rat. **a** showing normal and intact colonic wall that is mainly consisted of normal tunica mucosa (arrow) having folds of tubular glands or crypts lined by columnar epithelium rich with goblet cells, submucosa of highly vascularized connective tissue (dashed arrow), well-defined muscularis (M) and serosa (arrow head). **b**-**f** Colon of positive control rat treated with DSS, **b**) showing sever degenerations of the covering epithelium (arrow), noticeable diffuse leukocytic infiltration extending in between the mucosa and submucosa (inset box). **c** Higher magnification of fig **b** inset box showing the diffuse leukocytic infiltration (arrow head). **d**,**e** showing sever desquamations of the covering epithelium (arrow) and degenerations of the gland lining epithelium (arrow head). **f** showing sever vascular dilatation with congestion in the inter glandular areas and hemosidriosis (arrow). Stain: Haematoxylin & Eosin (H&E). Scale bars: a,b,d = 900 μm; c,e = 300 μm; f = 40 μm

The intoxicated rats with DSS showed severe degenerations of the covering epithelium with noticeable diffuse leukocytic infiltration extending in between the mucosa and submucosa **(**Fig. 3b, c**)**, desquamations of the covering epithelium and degenerations of the intestinal gland lining epithelium **(**Fig. 3d, e**)**, and prominent vascular dilatation with congestion in the inter glandular areas and hemosiderosis **(**Fig. 3f**)**. Meanwhile the negative control group showing normal and intact colonic wall that is mainly consisted of normal tunica mucosa having folds of tubular glands or crypts lined by columnar epithelium rich with goblet cells, submucosa of highly vascularized connective tissue, then well-defined muscularis and serosa **(**Fig. 3a**)**.

**Fig. 4:**
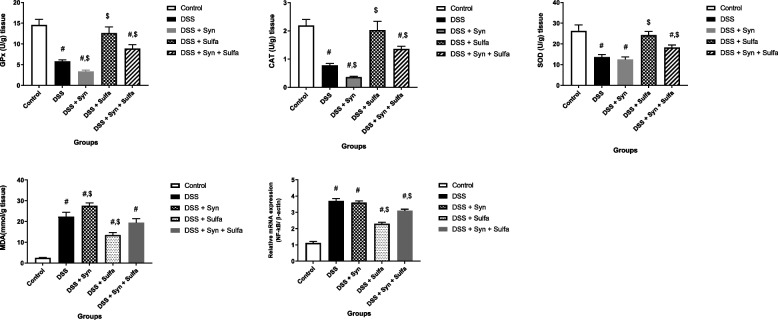
Effect of synbiotic (B.licheniformis and yeast extract) and or sulfasalazine (100 mg/kg) on GPx (U/g tissue), CAT (U/g tissue), SOD (U/g tissue), MDA (mmol/g tissue) and NF-kB/β-actin (relative expression) levels in colonic tissue against DSS induced colitis in rats. Data are expressed as the mean ± SEM. # indicates significant difference (*P* < 0.05) compared to control group. $ indicates *P* < 0.05 compared to DSS group (*n* = 10 treatments)

**Fig. 5:**
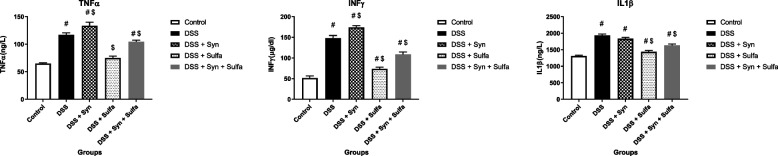
Effect of synbiotic (B.licheniformis and yeast extract) and or sulfasalazine (100 mg/kg) on proinflammatory cytokines TNFα (ng/L), INFγ (μg/dL), and IL1β (ng/L) level in serum against DSS induced colitis in rats. Data are expressed as the mean ± SEM. # indicates significant difference (*P* < 0.05) compared to control group. $ indicates *P* < 0.05 compared to DSS group (*n* = 10 treatments)

The DSS clearly amplified the colonic tissue MPO by 288% more than the untreated rats (Fig. 2). In addition, DSS induced statistical significant depression (*p* < 0.05) of intestinal tissue levels of SOD, CAT, and GPx (2.5, 2.2, and 1.9-fold changes), while it augmented the production of colonic MDA by 8.9 folds when compared with the control group, respectively (Fig. 4). The results of this study revealed that the experimental induction of colitis using DSS markedly upregulated the expression by 3.3 folds change for NF-kB than the control group (Fig. 4) and induced a significant increase (*P* < 0.05) in the serum levels of TNFα (116.91 ± 3.74), INFγ (148.03 ± 6.37), and IL-1β (1931.55 ± 44.91) in comparison to the control group; TNFα (65.12 ± 1.322), INFγ (51.57 ± 51.57), and Il-1β (1309.65 ± 28.02), respectively as shown in Fig. 5.

The current findings demonstrated that the tested synbiotic raised all inflammatory markers and deteriorated the DSS induced colitis where the DAI of this group (Fig. 1) indicated that the first clinical signs of colitis in the acute study were at the 2nd day after starting DSS administration (hemoccult+), whereas at the fourth day some animals started to present gross bleeding (score 4). The presence of blood in feces and differences in weight loss during the disease induction process were most pronounced in the last day of DSS-colitis induction.

Figures 2 and 4 presented that the WBCs count, intestinal MPO level and NF-κB expression of (DSS + Syn) group were significantly increased (*P* < 0.05) than the control group (4.16- and 3.2-fold changes). The oxidative stress parameters: CAT, SOD, GPx, and MDA, in the colonic tissue of DSS + Synbiotic treated rats were noticeably deteriorated (Fig. 4). In addition, the synbiotic administration markedly elevated serum levels of the pro-inflammatory cytokines; TNF α (2.05 folds), INFγ (3.38 folds), and IL1-β (1.4 folds) compared to the control group and TNF α (1.14 folds), INFγ (1.18 folds) than the DSS group (Fig. 5).

**Fig. 6:**
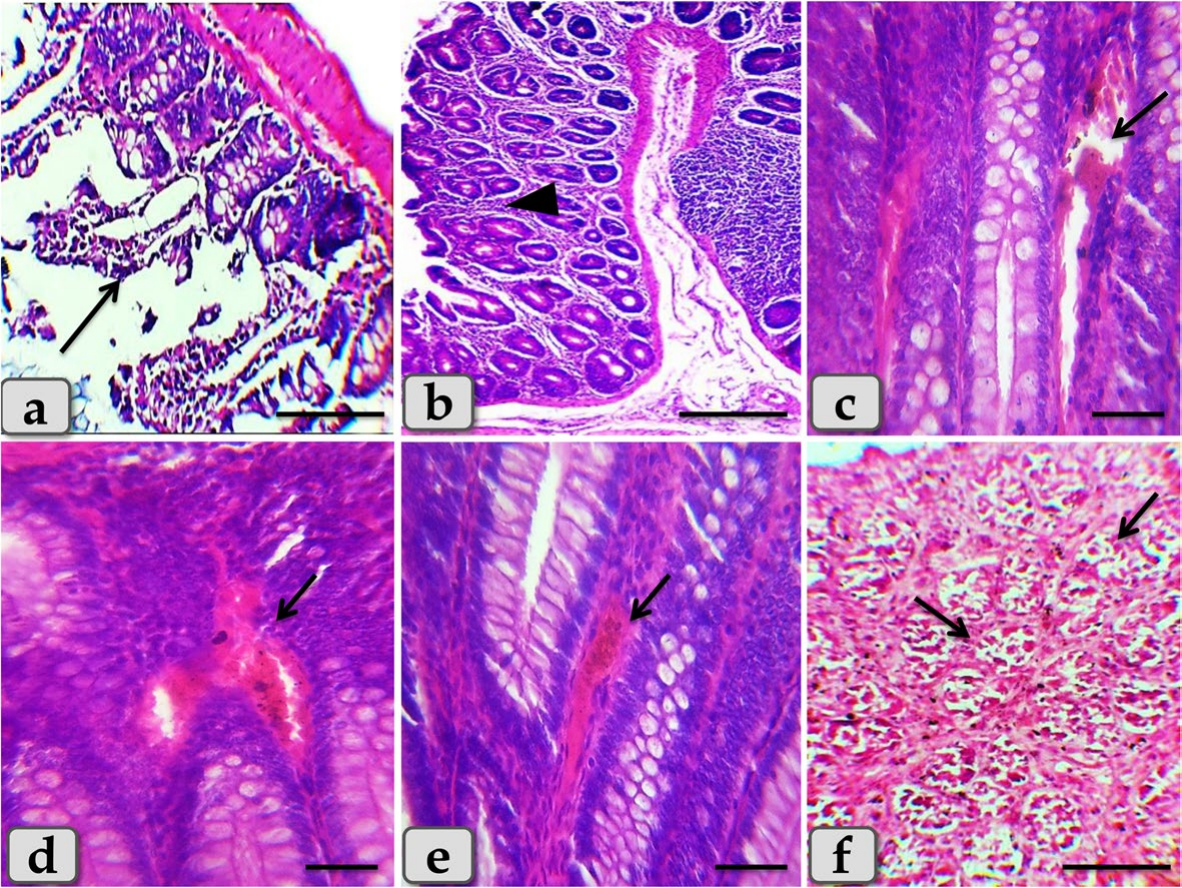
Colon of the DSS + Syn treated group, **a**) showing sever degenerations and desquamations of the covering epithelium with loss of the cells details (arrow). **b** showing diffuse inflammatory cells infiltrations in the inter glandular areas of the mucosa (arrow head). **c** showing sever blood vessels dilatation with congestion (arrow). **d** showing sever hemorrhage in the inter glandular areas (arrow). **e** showing moderate hemosiderosis (arrow). **f** showing diffuse coagulative necrosis of the entire intestinal mucosa characterized with maintaining tissue architecture but with completely loss of cellular details of some examined sections (arrow). Stain: H&E. Scale bars: All = 300 μm, except c, d, e = 40 μm

On the histological level the synbiotic treatment produced intense degenerations and desquamations of the covering epithelium with loss of the cell’s details **(**Fig. 6a**)**, severe catarrhal enteritis represented in diffuse inflammatory cells infiltration in the mucosa, congestion in the inter glandular area with goblet cells hyperplasia with excessive mucous secretions filling the intestinal glands **(**Fig. 6b, c**)**, hemorrhage **(**Fig. 6d**)**, and moderate hemosiderosis **(**Fig. 6e**)**. In addition, ischemic colitis with diffuse coagulative necrosis of the entire intestinal mucosa characterized by maintaining tissue architecture but with completely loss of cellular details was also clarified of some examined sections (Fig. 6f**)**.

Among DSS-colitis groups, it is remarkable that the group of rats that administered the sulfasalazine exhibited fewer changes in the symptoms evaluated during the induction period. The DAI of this group exhibited the lowest weight loss, regular stool consistency until the 4th day and the presence of slight bleeding either occult or apparent in the stool only on the 6th and 7th days (Fig. 1). The pro-inflammatory cytokines were significantly reduced (*P* < 0.05) by 36% for TNFα, 50% for INFγ, and 26% for IL-1β from the DSS group (Fig. 5). Figure 2 presented that the WBCs count, MPO level, were also obviously decreased than that of the DSS group by 40 and 64.7%, respectively. The antioxidant enzymes: GPx, SOD, and CAT, were markedly augmented, while the MDA was obviously depressed when compared with the DSS group.

**Fig. 7:**
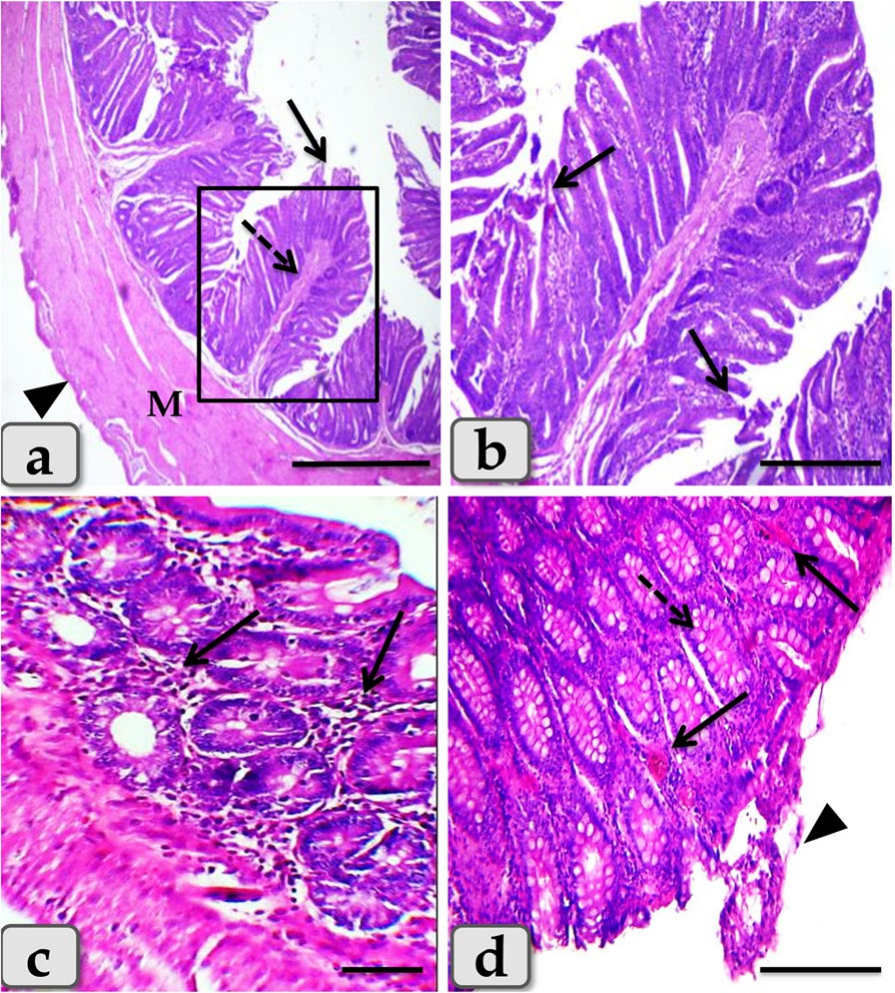
Colon of the DSS + Sulfa treated group, **a**) showing normal, intact colonic wall; mucosa (arrow), submucosa (dashed arrow), musculosa (M) and serosa (arrow head). **b** showing intact mucosa but with mild degeneration and desquamation of the covering epithelium (arrow). **c** showing mild to moderate inflammatory cells infiltrations in the mucosa (arrow). **d** showing hyperplastic changes of the intestinal gland & goblet cells hyperplasia with excessive mucous secretions (dashed arrow), minute hemorrhage (arrow), in addition, focal area of degeneration and desquamation of the covering epithelium (arrow head). Stain: H&E. Scale bars: a = 900 μm; b,d = 300 μm; c = 40 μm

Sulfasalazine treatment ameliorated the pathological damage in the colon induced with DSS and demonstrated mild degeneration and desquamation of the covering epithelium **(**Fig. 7a, b**)**, mild to moderate inflammatory cells infiltrations in the mucosa **(**Fig. 7c**)**, hyperplastic changes of the intestinal gland and its lining goblet cells with excessive mucous secretions, and minute hemorrhage **(**Fig. 7d**)**.

**Fig. 8:**
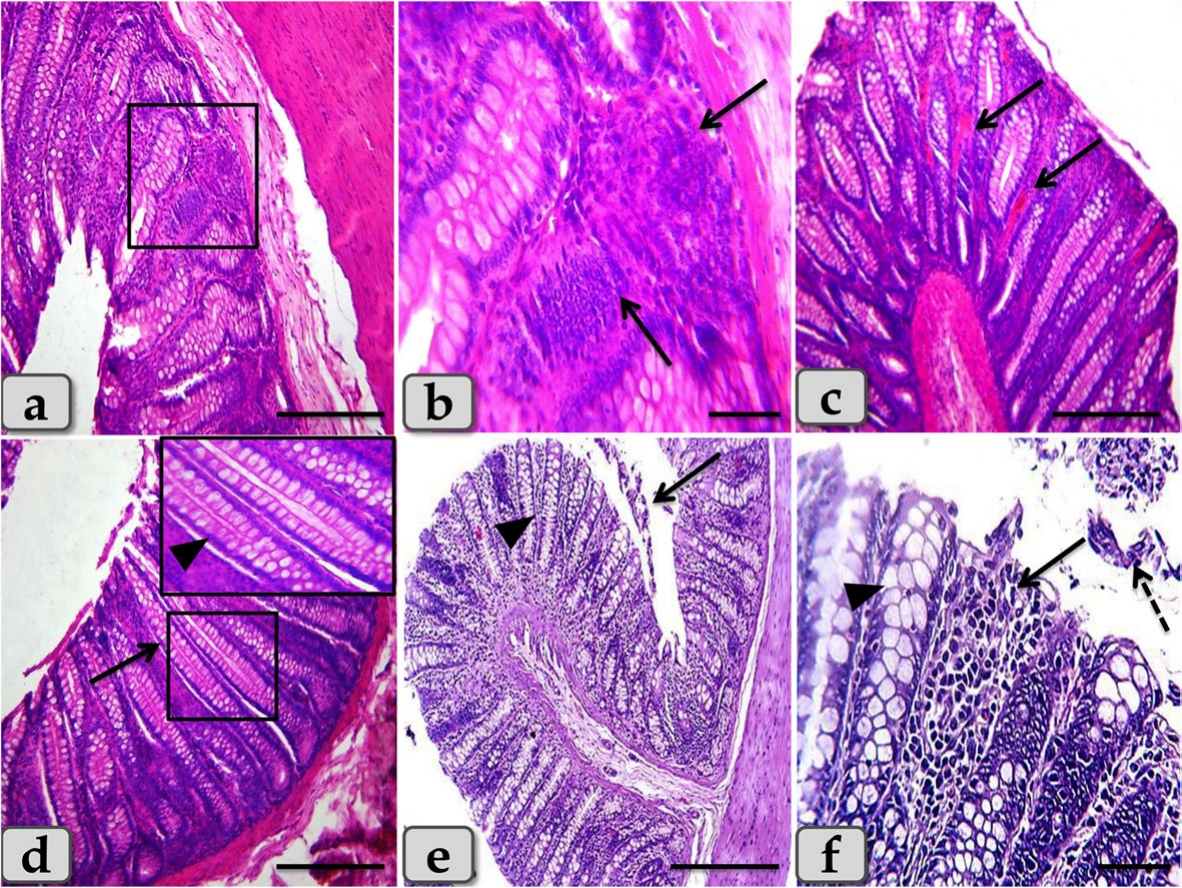
Colon of the DSS + Syn + Sulfa treated group **a**&**b**) showing moderate lymphocytic aggregations in the mucosa (arrow). **c** showing moderate vascular congestion (arrow) and minute hemorrhage. **d** showing massive hyperplastic changes of the entire intestinal glands (arrow) with goblet cells hyperplasia with excessive mucous secretions (arrow head). **e** showing massive intestinal glands hyperplasia (arrows head) and desquamation of the covering epithelium (arrow). **f** showing goblet cells hyperplasia of the intestinal glands (arrowhead), with diffuse mononuclear cells infiltrations (arrow) and moderate desquamation of covering epithelium (dashed arrow). Stain: H&E. Scale bars: All = 300 μm except b, f = 40 μm

Furthermore, the sulfasalazine reduced the inflammatory condition caused by the tested synbiotic and DSS in (DSS + Syn + Sulfa) group. This group presented moderate DAI (Fig. 1), proinflammatory cytokines profile (Fig. 5), MPO level (Fig. 2), oxidative parameters level (Fig. 4), NF-κB expression (Fig. 4) and Grade II (Fig. 3c) pathological lesion including colitis with showing massive coagulative necrosis of the entire intestinal mucosa. Moreover, colon sections of this group revealed moderate lymphocytic aggregations in the mucosa (Fig. 8a, b), moderate vascular congestion and minute hemorrhage (Fig. 8c). Also massive hyperplastic changes of the entire intestinal glands & goblet cells with diffuse mononuclear cells infiltrations were also observed with moderate desquamation of covering epithelium **(**Fig. 8d, e, f**)**.

## Discussion

Probiotics and/or prebiotics have been evaluated for the management of some of gastrointestinal disorders including UC [37, 38], pouchitis [38] and CD [39, 40]. Since the safety profile of probiotics and prebiotics in patients with IBD remains less explored, the current study investigated the potential for the synbiotic mixture of *Bacillus licheniformis* DSM 17236 and an extracted prebiotic derived from cell walls of the baker yeast *Saccharomyces cerevisiae* (It distinguishes itself by high amounts of Mannan-Oligosaccharides (MOS) and ß-Glucans), in comparison to sulfasalazine regarding their role on the severity of DSS-induced colitis in Sprague Dawley rats.

It was found that the synbiotic combination was not able to reduce the symptomatic, heamatological, biochemical and histopathological features of DSS colitis. Interestingly, synbiotic treatment actually increased most of the tested inflammatory indicators of DSS-colitis.

In our study, DSS intoxication for 7 days developed the symptoms of the inflammatory conditions in colon, markedly increased the DAI with the 2nd day, and elevated the WBCs count in blood. These findings are similar to what was mentioned by other authors who had applied the same way of induction [20, 41, 42].

DSS model for induction of ulcerative colitis was originally reported by Okayasu et al. [43]. DSS-induced colitis is becoming a fixed model that is phenotypically similar to ulcerative colitis in humans [44]. Oral administration of DSS for several days, leads to enteric and colonic epithelial lesions including ulcerations, detached mucosa, epithelial erosions and acute inflammation characterized by the presence of RBCs, neutrophils and macrophages within damaged segments [9, 20, 45]. This was in accordance with the severe necrotizing colitis demonstrated in our histopathological results. Therefore, the acute DSS colitis model is particularly applicable to study probiotic effects on epithelial barrier function.

The main histological feature of colitis is the leukocytic infiltration of the colonic tissue specially with the polymorph nuclear cells and monocytes [9, 46]. Neutrophiles migrate to the damaged colonic tissue during the acute inflammation and is associated with elevated MPO activity as a parameter reflecting the severity of the lesion in acute colitis [47, 48]. This was in accordance with our findings where the DSS intoxication clearly amplified the colonic tissue MPO more than the untreated rats. MPO has a peroxidase activity that produce hypochloric acid from hydrogen peroxide that in turn induce the release of free oxygen radicals from activated neutrophils [49].

The imbalance between the increased amounts of toxic reactive oxygen and nitrogen species (ROS and RNS) and the capacity of the tissue antioxidant defense mechanisms; CAT, SOD, and GPx, leads to oxidative injury and increases mucosal injury in colitic patients [48, 50]. The ROS and RNS trigger the damage of protein, nucleic acids, and lipid peroxidation of colonic mucus membranes [51]. Elevated MDA is the associated marker to lipid peroxidation of the colonic tissue [52, 53]. In our work DSS depressed the intestinal tissue levels of SOD, CAT, and GPx, while augmented the production of colonic MDA.

The free radicals and the cytokine like property of MPOinduces nuclear factor NF-κB which is a transcription factor that controls the expression of genes of the proinflammatory cytokine; TNF-α, IFN-γ and IL-1β [54]. Increased expression of NF-κB factor has been associated with the pathogenesis of IBD [55]. In accordance, our findings revealed that the experimental induction of colitis using DSS markedly upregulated the expression for NF-kB than the control group.

Despite the DSS-colitis is caused through the toxic damage of the intestinal and colonic epithelial cells, the inflammatory reactions ensue after the proinflammatory cytokines (Il-1β, Il-6, TNFα, and INFγ) production by tissue macrophages that seems to play an essential role in the disease process [56, 57]. In this study, the DSS intoxication induced a significant raise in the serum levels of TNFα, INFγ, and IL-1β.

TNF-α exerts its pro-inflammatory impacts via the raised production of IL-1β and IL-6, as well as initiation of cytotoxic and apoptotic response [58–60]. TNF-α production by human macrophages was discovered in the colonic tissue in patients with CD and UC [61], and there is linear correlation between serum levels of TNF-α with clinical and laboratory indices of intestinal disease activity [62]. IFN-γ and IL-1 family of cytokines have eminent roles in IBD pathogenesis [63–65]. In UC, IL-1 promotes inflammation where it is secreted from monocytes and macrophages, and activated into IL-1β in the colonic mucosa [66]. IFN-γ, produced from the colonic lamina propria mononuclear cells (LPMC), causes disruption of the vascular barrier and increases the permeability of colonic tissue. INF-γ is reduced as a result of anti-TNF-α treatments [63]. The current findings showed that the tested synbiotic raised the DAI scoring, WBCs count, intestinal MPO level and NF-κB expression. The oxidative stress parameters; CAT, SOD, GPx, and MDA, in the colonic tissue of DSS + Synbiotic treated rats were noticeably deteriorated. In addition, the synbiotic administration markedly elevated serum levels of the pro-inflammatory cytokines; TNF α, INFγ, and IL1-β.

On the histological level, the synbiotic treatment induced severe damage of the covering epithelium, coagulative necrosis, and catarrhal enteritis.

These results were in contrary to Li Y et al. who reported that *Bacillus licheniformis* Zhengchangsheng® succeeded to attenuate the severity of colitis induced by DSS in mice where it reduced the weight loss, colonic mucosal damage and increased the DAI [67]. Moreover, widely reported evidences indicated that the use of single probiotic; *Bifidobacterium* [68], or *Lactobacillus* strains [69], or mixtures of probiotics; quadruple probiotic mixture (P-qua), Siliankang, consisting of *Bifidobacterium infantis, Lactobacillus acidophilus, Enterococcus faecalis, and* aerobic *Bacillus cereus* [70], or VSL#3 which contains eight bacterial species including four strains of lactobacilli, three strains of Bifidobacteria and *Streptococcus salivarius subsp. thermophiles* [71–74], have shown potential capabilities to decrease the pro-inflammatory cytokines, mucosal ulcerations, leukocytic infiltrations in the damaged tissue, and in general ameliorated the acute and chronic DSS colitis in a preventive and a therapeutic regimens. Yeast cell wall MOS was administered in DSS-induced mouse model of acute colitis and found to reduce the DAI and histological scores (mucosal damage) [36]. Inulin and beta glucans have been demonstrated to reduce the inflammation symptoms in patients with IBD [75, 76].

On the other hand, because probiotics have influences on both the innate and acquired immune systems, including effects on cytokine secretion and dendritic cell function [77, 78], exaggerated concerns have been raised about the potential to overly stimulate the immune response in some individuals, possibly leading to inflammation or autoimmune phenomena [79].

In accordance with our work, feeding chicks with *Bacillus subtilis* Gallipro DSM 17299 and MOS depressed the growth rate and elevated serum C-reactive protein (CRP), acute phase protein, that is enhanced in response to stimulation by both IL-1 and IL-6, such increase reflects intestinal inflammatory process [80, 81]. Adding *Bacillus licheniformis* to diet amplified mRNA expression of NF-κB in the jejunum of broilers and in turn activation of their signaling cascade leading to initiation of cellular responses of innate immune cells and production of proinflammatory cytokines [82]. Porcine intestinal epithelial cells (IPEC-J2 cells) treated with *Bacillus licheniformis* showed increased production of the proinflammatory cytokine, IL-8, significantly more than the control group, while *Bacillus subtilis* increased IL-6 secretion [83]. This indicate that normal microbiota might increase proinflammatory cytokines similar to pathogen induced response as detected in other studies [84].

In the same line, Zhou et al. demonstrated that animals treated with *Lactobacillus crispatus* M206119 suspension induced a higher severity of colitis in the form of elevated DAI involving diarrhea, bloody stools, greater weight loss, higher histopathological damage and leukocytic infiltration when compared to the control group [85]. Moreover, *Lactobacillus salivarius subsp. salivarius* 433,118 (UCC118) did not have a beneficial effect on DSS colitis or on pathological or physiological parameters [86]. Other strains like *L. acidophilus* NCFM, *L. planetarium* NCIMB8826 and *L. rhamnosus* GG also did not attenuate but deteriorate some colitis symptoms [87–89].

In a double-blind placebo-controlled trial, Mangalat et al. observed an eminent proinflammatory triggering following the administration of *Lactobacillus reuteri* in healthy adults and increased fecal calprotectin [90]. Moreover, some individuals taking probiotic may temporarily experience an increase in gas production and swelling, in addition to constipation [91, 92].

There are numerous clinical and experimental studies that yeast might induce intestinal inflammation. Anti–*Saccharomyces cerevisiae* antibodies (ASCAs) was discovered in the serum of CD patients [93, 94] which indicates that an aberrant immune reaction to yeast might be engaged in IBD progression. Experimentally, treatment with yeast worsened the DAI in DSS colitis and exacerbated ASCA levels, in addition promoted serum and colonic tissue pro-inflammatory cytokine secretion (TNF- α, IL-1β and IL-6) and stimulated the expression of NF-κB in colonic tissue [95]. In the same line, Chiaro et al., reported that animals treated with *Saccharomyces cerevisiae* exhibited intestinal epithelial damage, more crypt loss, and inflammatory cells infiltration [96].

Interestingly, FOS treatment, alone and in synbiotic combination with *L. fermentum* BR11, elevated the rigorousness of some indicators of DSS-colitis [32]. β-glucans increased intestinal inflammation and inflammatory cytokines level in colonic tissue after DSS-colitis [97]. Similarly, zymosan (a product derived from *Saccharomyces cerevisiae* and contains about 55% β-glucans) aggravated the progression of DSS-induced colitis and amplified colonic levels of pro-inflammatory cytokines [97].

The interactions of different oligosaccharides (MOS, FOS and inulin) with different carbohydrate receptors located on either epithelial or immune cells can trigger the immune reactions in the gastrointestinal tract including the inflammation [98, 99]. We can in ease say that the probiotic strain, prebiotic type, in addition to the duration and dose may play a central role in modulating the inflammatory colitis.

Sulfasalazine, the union of 5-aminosalicylic acid (biologically active molecule) and sulfapyridine moiety, is an analgesic and non-steroidal anti-inflammatory drug (NSAID) from the salicylates group, which has been extensively used for the management of moderate ulcerative colitis [54, 100, 101]. It acts through suppressing the migration of polymorphonuclear leukocytes to intestinal wall, as well as inhibits macrophages to produce IL-1β, proinflammatory leukotrienes (LTB4 and 5-HETE), and prostaglandins [49, 101–104]. This was supported with our results where sulfasalazine treated rats showed fewer changes in the symptoms, WBCs count, pro-inflammatory cytokines, tissue MPO, NF-κB expression, oxidative stress markers and histopathological level in comparison to the control group. This was in agreement with the results of some researches which reported that sulfasalazine administration led to anti-inflammatory effects including reduced body weight loss, proinflammatory cytokines level, MPO level, NF-κB expression, and microscopic damage induced by DSS intoxication [54, 105, 106].

However, there is a lack of availability to address some key points in the current study regarding exploring the colitis associated and the effect of the tested symbiotic on T-cell clonotypes through analyzing the colonic T cell receptor repertoire of the diseased, synbiotic treated, and control animals. The potential implication of immunophenotyping of T cell subsets associated with colitis needs further investigations in the future.

## Conclusion

We can conclude that synbiotic mixture of *Bacillus licheniformis* DSM 17236 and an extracted prebiotic derived from cell walls of the baker yeast *Saccharomyces cerevisiae* does not reduce but increased the severity of experimentally-induced colitis in Spargue dawley rats. Future studies could investigate the effects of this synbiotic on the inflammatory reactions in the gut, which may affect the extent of colonic injury. The application of probiotics and/or prebiotics as a potential adjunctive treatment for IBD remains controversial and further experimental studies are indicated.

## Methods

### Chemicals and kits

Sulfasalazine® was purchased from Rosemont Pharmaceuticals Co., UK, and dextran sulfate sodium (DSS) from MP Biomedicals Co., UK. Commercial, double sandwich, enzyme-linked immunosorbent assays (ELISA) kits for the accurate quantification of Interferon γ (IFN-γ) with Cat. No. E0105Hu, Interleukin 1β(IL-1β) with Cat. No. E0094Hu and Tumor necrosis factor α (TNF-α) with Cat. No. E0082Hu in serum. These ELISA kits were obtained from Bioassay Technology Laboratory Co., China. MDA diagnostic kit was purchased from Biodiagnostics Co. (Cairo, Egypt), while the MPO kit was bought from Jiancheng biotech Co (China). All other chemicals were purchased from standard commercial suppliers and were of analytical grade.

### Synbiotic preparations

*Bacillus licheniformis* DSM 17236 (GalliPro® Tect) is purchased from (Biochem company, Germany). This probiotic strain has been isolated from soil and is a non-genetically modified organism (GMO). *Bacillus licheniformis* are Gram positive organisms, Spore formers and facultative aerobe but at presence of nitrate/nitrite it can grow anaerobic. The product was mixed at a final concentration of 1.6 × 10^9^ CFU/kg of the basal diet [107].

TechnoMos® (Biochem company, Germany**)** is an extracted prebiotic derived from cell walls of the baker yeast *Saccharomyces cerevisiae.* It distinguishes itself by high amounts of Mannan-Oligosaccharides (MOS) and ß-Glucans. It is mixed at 0.1% (1 g/kg) of the basic diet [108].

### Rats and ethical statement

Fifty adult male Sprague Dawley rats (200–250 g) were used. The age of rats was about 8 months. They were obtained from the laboratory animal unit, Faculty of Veterinary Medicine, Misurata University. They were clinically healthy, kept under hygienic condition and housed in plastic cages with hard wood shavings as bedding and at a controlled temperature (21–24 °C) with a relative humidity of 50–60% and a 12-hr light-dark cycle. They were fed a balanced diet and had free access to clean water. All animals were accommodated for laboratory conditions for 2 weeks before being experimented. Animals were managed according to the Guidelines for the Care and Use of Laboratory Animals of the Institutional Animal Care and Use Committee of the Zagazig University, Egypt (**ZU-IACUC**), and Approval number (***ZU-IACUC/2/F/31/2022***) and all methods are reported in accordance with ARRIVE guidelines.

### Experimental protocol

The rats were simply randomized and allocated into five groups of ten animals in each group and the experimenter followed single blind design during the experiment and analysis:

- Control group: healthy animals that were fed on basal diet.
- DSS group: animals with DSS induced ulcerative colitis and fed on basal diet only.
- DSS + Syn group: animals with chemically induced ulcerative colitis and fed on the synbiotic combination that was mixed with the basal diet.
- DSS + Sulfa group: animals with chemically induced ulcerative colitis, treated with intragastric sulfasalazine (100 mg/kg of body weight) and fed on the basal diet.
- DSS + Syn + Sulfa group: animals with chemically induced ulcerative colitis, treated with intragastric sulfasalazine (100 mg/kg) and fed with the synbiotic combination that was mixed with the normal basal diet.

As described previously [109] ulcerative colitis was induced by dissolving 3.5% DSS in the animals’ drinking water; this solution is prepared every day for a period of successive 7 days. Throughout the experimental period, individual body weight and fecal output were examined daily. The severity of DSS-colitis was determined using the disease activity index (DAI) that depends on the methodology of Murthy et al. [110] who uses a scoring system taking into account three parameters: weight loss, stool consistency, rectal bleeding in feces where each criteria was given a score between 0 and 4, thus resulting in the total DAI score. The DAI was used as an indicator of rat health between Days 7 and 14 of the experiment. Synbiotic was fed to animals 7 days prior to, along with and 14 days after chemical induction of ulcerative colitis. Sulfasalazine was intragastrically given to animals 14 days after the induction of the colitis. After the last treatment, animals were euthanized using halothane. Gastrointestinal samples were prepared for biochemical, RT-PCR and histopathological examinations. Whole blood was collected in tubes with EDTA, while serum was separated after collecting the blood without anticoagulant then centrifugation in 5000 G for 10 min.

### Cytokines assay

Detection of serum TNFα, INFγ, and IL-1-β using double antibodies sandwich ELISAs were adopted for estimation of the three cytokines according the manufacturer’s guidelines.

### WBCs count

A sample volume of a whole blood specimen is aspirated into the analyzer. M- 52DIFF LYSE applies to BC-5000 Auto Hematology Analyzer manufactured by Mindray to dissolve the WBCs for their counting according to the manufacturer’s instructions.

### MPO activity

MPO was measured in the homogenate supernatant of the intestinal tissue as a marker of neutrophile accumulation in colonic mucosa as preciously described [111].

### MDA activity

Lipid Peroxides (MDA) was estimated in intestinal tissue homogenate according to instructions described in the kits and the method of [51].

### The transcriptional levels of necrotic factor kappa B (NF-κB) in intestinal tissue

Total RNA was extracted from 20 mg of colon tissues using the RNeasy kit of Qiagen (Cat. No.74104). The high purity of the extracted RNA samples were confirmed using a NanoDrop spectrophotometer technologies (Wilmington, Dela-ware, USA). The gene-specific primer sets used in the present study were as follows: -kB primers, F:5ˋ-AAT TGC CCC GGC AT -3ˋ, R: 5ˋ- TCC CGT AACCGC GTA -3ˋ (Accession No. XM_342346.4) and β-actin primers, F:5ˋ- TCA CTA TCGGCAATGTGC GG -3ˋ, R: 5ˋ- GCT CAG GAG GAG CAA TGA TG -3ˋ (Accession No.NM_007393). cDNA was prepared by the reverse transcription reaction (QIAamp RNA Mini Kit (Qiagen, Germany, Gmbh). The real-time PCR reaction was performed in a total volume of 20 μl of the following reaction mixture: 1 μl of each primer (20 pmol), 6 μl template DNA, 10 μl SYPR® Green (1X) and 2 μl water nuclease-free. The samples were transferred to each well of a PCR plate. Rotor-Gene Q2 plex (Qiagen Inc., Valencia, CA, USA) was used to carry out the amplification reaction. The relative quantitation detection method used for calculating the transcripts of the replicates and β-actin was used as a house keeping gene for normalization [112].

### Histopathology

Specimens of colon were collected and immediately fixed in 10% neutral buffered formalin for 48 hrs and then processed histologically. These specimens were dehydrated in ascending grades of ethanol, cleared in xylene and embedded in paraffin wax forming paraffin blocks. 4–5 μm thick sections were obtained and stained with Harris Hematoxylin and Eosin (H&E) [113]. The microphotographs were taken using a digital Dsc-W130 super steady cypershot camera (Sony, Japan) connected to an Olympus BX 21 light microscope.

### Statistical analysis

All data were tested for normality by the Shapiro-Wilk test and homogeneity of variance by Levene’s mean test before being analyzed with a one-way ANOVA followed by a Tukey’s multiple-comparison test using SPSS/21 software. The figures were generated using GraphPad Prism 7.0. The data were expressed as mean ± standard error of mean (SEM) for ten animals in each group. *P* values of less than 0.05 were considered to indicate statistical significance.

## Data Availability

The datasets used the present study are available from upon request the corresponding author.

## Abbreviations

GPx: Glutathion peroxidase
CAT: Catalase
SOD: Superoxide dismutase
TNFα: Tumor necrotic factor alfa
INFγ: Interferon gamma
IL-1β: Interleukin 1-beta
NF-kB: Nuclear factor kappa B

## References

[1] Colombel JF, Mahadevan U (2017). Inflammatory bowel disease 2017: innovations and changing paradigms. Gastroenterology.

[2] Kelsen JR, Russo P, Sullivan KE (2019). Early-onset inflammatory bowel disease. Immunol Allergy Clin N Am.

[3] Dotan I, Rachmilewitz D (2005). Probiotics in inflammatory bowel disease: possible mechanisms of action. Curr Opin Gastroenterol.

[4] Ohman L, Simrén M (2010). Pathogenesis of IBS: role of inflammation, immunity and neuroimmune interactions. Nat Rev Gastroenterol Hepatol.

[5] Salim SY, Söderholm JD (2011). Importance of disrupted intestinal barrier in inflammatory bowel diseases. Inflamm Bowel Dis.

[6] Arab HH, Salama SA, Eid AH, Omar HA, Arafa E-SA, Maghrabi IA (2014). Camel’s milk ameliorates TNBS-induced colitis in rats via downregulation of inflammatory cytokines and oxidative stress. Food Chem Toxicol.

[7] Lin L, Wang D, Qu S, Zhao H, Lin Y (2020). miR-370-3p alleviates ulcerative colitis-related colorectal Cancer in mice through inhibiting the inflammatory response and epithelial-mesenchymal transition. Drug Des Devel Ther.

[8] Wirtz S, Neurath MF (2007). Mouse models of inflammatory bowel disease. Adv Drug Deliv Rev.

[9] Huang Y, Qiu L, Mi X, Zhang Z, Xu D, Tao X, et al. Hot-water extract of ripened Pu-erh tea attenuates DSS-induced colitis through modulation of the NF-κB and HIF-1α signaling pathways in mice. Food Funct. 2020. 10.1039/c9fo02803j. 10.1039/c9fo02803j 32239008

[10] Fang J, Seki T, Tsukamoto T, Qin H, Yin H, Liao L (2013). Protection from inflammatory bowel disease and colitis-associated carcinogenesis with 4-vinyl-2,6-dimethoxyphenol (canolol) involves suppression of oxidative stress and inflammatory cytokines. Carcinogenesis.

[11] Pawar P, Gilda S, Sharma S, Jagtap S, Paradkar A, Mahadik K, et al. Rectal gel application of Withania somnifera root extract expounds anti-inflammatory and muco-restorative activity in TNBS-induced inflammatory bowel disease. BMC Complement Altern Med. 2011;11–34. 10.1186/1472-6882-11-34 PMC3103490 21527003

[12] Achitei D, Ciobica A, Balan G, Gologan E, Stanciu C, Stefanescu G (2013). Different profile of peripheral antioxidant enzymes and lipid peroxidation in active and non-active inflammatory bowel disease patients. Dig Dis Sci.

[13] Cadirci E, Suleyman H, Aksoy H, Halici Z, Ozgen U, Koc A (2007). Effects of Onosma armeniacum root extract on ethanol-induced oxidative stress in stomach tissue of rats. Chem Biol Interact.

[14] Moura FA, de Andrade KQ, dos Santos JCF, Araújo ORP, Goulart MOF (2015). Antioxidant therapy for treatment of inflammatory bowel disease: does it work?. Redox Biol.

[15] Rutgeerts P, Vermeire S, van Assche G (2009). Biological therapies for inflammatory bowel diseases. Gastroenterology.

[16] Barnes PJ, Adcock IM (2009). Glucocorticoid resistance in inflammatory diseases. Lancet.

[17] Ng SC, Lam YT, Tsoi KKF, Chan FKL, Sung JJY, Wu JCY (2013). Systematic review: the efficacy of herbal therapy in inflammatory bowel disease. Aliment Pharmacol Ther.

[18] Macho Fernandez E, Pot B, Grangette C (2011). Beneficial effect of probiotics in IBD: are peptidogycan and NOD2 the molecular key effectors?. Gut Microbes.

[19] Orel R, Trop TK (2014). Intestinal microbiota, probiotics and prebiotics in inflammatory bowel disease. World J Gastroenterol.

[20] Din AU, Hassan A, Zhu Y, Zhang K, Wang Y, Li T, et al. Inhibitory effect of Bifidobacterium bifidum ATCC 29521 on colitis and its mechanism. J Nutr Biochem. 2020;79:108353. 10.1016/j.jnutbio.2020.108353 32145470

[21] Hong HA, Le HD, Cutting SM (2005). The use of bacterial spore formers as probiotics. FEMS Microbiol Rev.

[22] Barbosa TM, Serra CR, La Ragione RM, Woodward MJ, Henriques AO (2005). Screening for Bacillus isolates in the broiler gastrointestinal tract. Appl Environ Microbiol.

[23] Spinosa MR, Braccini T, Ricca E, De Felice M, Morelli L, Pozzi G (2000). On the fate of ingested Bacillus spores. Res Microbiol.

[24] Sorokulova IB, Pinchuk IV, Denayrolles M, Osipova IG, Huang JM, Cutting SM (2008). The safety of two Bacillus probiotic strains for human use. Dig Dis Sci.

[25] Luise D, Bosi P, Raff L, Amatucci L, Virdis S, Trevisi P, et al. Probiotic strains as a potential tool for limiting the use of antibiotics, and improving the growth and health of pigs and chickens. Front Microbiol. 2022;13:801827. 10.3389/fmicb.2022.801827 PMC8859173 35197953

[26] Pan L, Zhao PF, Ma XK, Shang QH, Xu YT, Long SF (2017). Probiotic supplementation protects weaned pigs against enterotoxigenic Escherichia coli K88 challenge and improves performance similar to antibiotics. J Anim Sci.

[27] Kritas SK, Govaris A, Christodoulopoulos G, Burriel AR (2006). Effect of Bacillus licheniformis and Bacillus subtilis supplementation of ewe’s feed on sheep milk production and young lamb mortality. J Vet Med A Physiol Pathol Clin Med.

[28] Xu Y, Yu Y, Shen Y, Li Q, Lan J, Wu Y, et al. Effects of Bacillus subtilis and Bacillus licheniformis on growth performance, immunity, short chain fatty acid production, antioxidant capacity, and cecal microflora in broilers. Poult Sci. 2021;100(9):101358. 10.1016/j.psj.2021.101358 PMC8350532 34358955

[29] Wang Y, Du W, Lei K, Wang B, Wang Y, Zhou Y (2017). Effects of dietary Bacillus licheniformis on gut physical barrier, immunity, and reproductive hormones of laying hens. Probiotics Antimicrob Proteins.

[30] Paap PM, van der Laak JH, Smit JI, Nakamura N, Beynen AC (2016). Administration of Bacillus subtilis C-3102 (Calsporin®) may improve feces consistency in dogs with chronic diarrhea. Res Opin Anim Vet Sci.

[31] Bengmark S (2002). Gut microbial ecology in critical illness: is there a role for prebiotics, probiotics, and synbiotics?. Curr Opin Crit Care.

[32] Geier MS, Butler RN, Giffard PM, Howarth GS (2007). Prebiotic and synbiotic fructooligosaccharide administration fails to reduce the severity of experimental colitis in rats. Dis Colon Rectum.

[33] Cukkemane A, Kumar P, Sathyamoorthy B (2020). A metabolomics footprint approach to understanding the benefits of synbiotics in functional foods and dietary therapeutics for health, communicable and non-communicable diseases. Food Res Int.

[34] Koleva PT, Valcheva RS, Sun X, Gänzle MG, Dieleman LA (2012). Inulin and fructo-oligosaccharides have divergent effects on colitis and commensal microbiota in HLA-B27 transgenic rats. Br J Nutr.

[35] Furrie E, Macfarlane S, Kennedy A, Cummings JH, Walsh SV, O’Neil DA (2005). Synbiotic therapy (Bifidobacterium longum/synergy 1) initiates resolution of inflammation in patients with active ulcerative colitis: a randomised controlled pilot trial. Gut.

[36] Ferenczi S, Szegi K, Winkler Z, Barna T, Kovács KJ (2016). Oligomannan prebiotic attenuates immunological, clinical and behavioral symptoms in mouse model of inflammatory bowel disease. Sci Rep.

[37] Bibiloni R, Fedorak RN, Tannock GW, Madsen KL, Gionchetti P, Campieri M (2005). VSL#3 probiotic-mixture induces remission in patients with active ulcerative colitis. Am J Gastroenterol.

[38] Dang X, Xu M, Liu D, Zhou D, Yang W (2020). Assessing the efficacy and safety of fecal microbiota transplantation and probiotic VSL#3 for active ulcerative colitis: a systematic review and meta-analysis. PLoS One.

[39] Schultz M, Timmer A, Herfarth HH, Sartor RB, Vanderhoof JA, Rath HC (2004). Lactobacillus GG in inducing and maintaining remission of Crohn’s disease. BMC Gastroenterol.

[40] Hrdý J, Alard J, Couturier-Maillard A, Boulard O, Boutillier D, Delacre M (2020). Lactobacillus reuteri 5454 and Bifidobacterium animalis ssp. lactis 5764 improve colitis while differentially impacting dendritic cells maturation and antimicrobial responses. Sci Rep.

[41] Malago JJ, Nondoli H (2008). Sodium arsenite reduces severity of dextran sulfate sodium-induced ulcerative colitis in rats. J Zhejiang Univ Sci B.

[42] Ozaki K, Makino H, Aoki M, Miyake T, Yasumasa N, Osako MK (2012). Therapeutic effect of ribbon-type nuclear factor-κB decoy oligonucleotides in a rat model of inflammatory bowel disease. Curr Gene Ther.

[43] Okayasu I, Hatakeyama S, Yamada M, Ohkusa T, Inagaki Y, Nakaya R (1990). A novel method in the induction of reliable experimental acute and chronic ulcerative colitis in mice. Gastroenterology.

[44] Björck S, Jennische E, Dahlström A, Ahlman H (1997). Influence of topical rectal application of drugs on dextran sulfate-induced colitis in rats. Dig Dis Sci.

[45] Jing Y, Liu H, Xu W, Yang Q (2017). Amelioration of the DSS-induced colitis in mice by pretreatment with 4,4′-diaponeurosporene-producing Bacillus subtilis. Exp Ther Med.

[46] Villegas I, De La Lastra CA, Orjales A, La Casa C (2003). A new flavonoid derivative, dosmalfate, attenuates the development of dextran sulphate sodium-induced colitis in mice. Int Immunopharmacol.

[47] Lau D, Mollnau H, Eiserich JP, Freeman BA, Daiber A, Gehling UM (2005). Myeloperoxidase mediates neutrophil activation by association with CD11b/CD18 integrins. Proc Natl Acad Sci U S A.

[48] Zhou Q, Zhang W-X, He Z-Q, Wu B-S, Shen Z-F, Shang H-T (2020). The possible anti-inflammatory effect of Dehydrocostus lactone on DSS-induced colitis in mice. Evid Based Complement Alternat Med.

[49] Podolsky DK (2002). Inflammatory bowel disease. N Engl J Med.

[50] Zhou X, Liu H, Zhang J, Mu J, Zalan Z, Hegyi F (2019). Protective effect of Lactobacillus fermentum CQPC04 on dextran sulfate sodium–induced colitis in mice is associated with modulation of the nuclear factor-κB signaling pathway. J Dairy Sci.

[51] Salem GA, Shaban A, Diab HA, Elsaghayer WA, Mjedib MD, Hnesh AM (2018). Phoenix dactylifera protects against oxidative stress and hepatic injury induced by paracetamol intoxication in rats. Biomed Pharmacother.

[52] Güven A, Güven A, Gülmez M (2003). The effect of kefir on the activities of GSH-Px, GST, CAT, GSH and LPO levels in carbon tetrachloride-induced mice tissues. J Vet Med Series B.

[53] Güvenç M, Cellat M, Özkan H, Tekeli İO, Uyar A, Gökçek İ (2019). Protective effects of Tyrosol against DSS-induced ulcerative colitis in rats. Inflammation.

[54] Yousefi-Ahmadipour A, Ebrahimi-Barough S, Niknia S, Allahverdi A, Mirzahosseini-pourranjbar A, Tashakori M, et al. Therapeutic effects of combination of platelet lysate and sulfasalazine administration in TNBS-induced colitis in rat. Biomed Pharmacother. 2020;125:109949. 10.1016/j.biopha.2020.109949 32058216

[55] Meng Q, Wu W, Pei T, Xue J, Xiao P, Sun L (2020). miRNA-129/FBW7/NF-κB, a novel regulatory pathway in inflammatory bowel disease. Mol Ther Nucleic Acids.

[56] Dykens JA, Baginski TJ (1998). Urinary 8-hydroxydeoxyguanosine excretion as a non-invasive marker of neutrophil activation in animal models of inflammatory bowel disease. Scand J Gastroenterol.

[57] Neurath MF (2014). Cytokines in inflammatory bowel disease. Nat Rev Immunol.

[58] Begue B, Wajant H, Bambou JC, Dubuquoy L, Siegmund D, Beaulieu JF (2006). Implication of TNF-related apoptosis-inducing ligand in inflammatory intestinal epithelial lesions. Gastroenterology.

[59] Sanchez-Muñoz F, Dominguez-Lopez A, Yamamoto-Furusho JK (2008). Role of cytokines in inflammatory bowel disease. World J Gastroenterol.

[60] Xiao YT, Yan WH, Cao Y, Yan JK, Cai W (2016). Neutralization of IL-6 and TNF-α ameliorates intestinal permeability in DSS-induced colitis. Cytokine.

[61] Stucchi A, Reed K, O’Brien M, Cerda S, Andrews C, Gower A (2006). A new transcription factor that regulates TNF-α gene expression, LITAF, is increased in intestinal tissues from patients with CD and UC. Inflamm Bowel Dis.

[62] Zhou Q, Shen Z-F, Wu B, Xu C, He Z, Chen T, et al. Risk of colorectal Cancer in ulcerative colitis patients: a systematic review and Meta-analysis. Gastroenterol Res Pract. 2019;2019:5363261. 10.1155/2019/5363261 PMC6874962 31781191

[63] Langer V, Vivi E, Regensburger D, Winkler TH, Waldner MJ, Rath T (2019). IFN-γ drives inflammatory bowel disease pathogenesis through VE-cadherin-directed vascular barrier disruption. J Clin Invest.

[64] Dinarello CA (2002). The IL-1 family and inflammatory diseases.

[65] Dinarello CA (2009). Interleukin-1β and the autoinflammatory diseases. N Engl J Med.

[66] McAlindon ME, Hawkey CJ, Mahida YR (1998). Expression of interleukin 1β and interleukin 1β converting enzyme by intestinal macrophages in health and inflammatory bowel disease. Gut.

[67] Li Y, Liu M, Zhou J, Hou B, Su X, Liu Z (2019). Bacillus licheniformis zhengchangsheng® attenuates DSS-induced colitis and modulates the gut microbiota in mice. Benef Microbes.

[68] Li S-C, Hsu W-F, Chang J-S, Shih C-K (2019). Combination of Lactobacillus acidophilus and Bifidobacterium animalis subsp. lactis shows a stronger anti-inflammatory effect than individual strains in HT-29 cells. Nutrients.

[69] Wang G, Liu Y, Lu Z, Yang Y, Xia Y, Lai PF-H (2019). The ameliorative effect of a Lactobacillus strain with good adhesion ability against dextran sulfate sodium-induced murine colitis. Food Funct.

[70] Chen Y, Zhang L, Hong G, Huang C, Qian W, Bai T (2020). Probiotic mixtures with aerobic constituent promoted the recovery of multi-barriers in DSS-induced chronic colitis. Life Sci.

[71] Chapman TM, Plosker GL, Figgitt DP (2006). VSL#3 probiotic mixture: a review of its use in chronic inflammatory bowel diseases. Drugs.

[72] Rachmilewitz D, Katakura K, Karmeli F, Hayashi T, Reinus C, Rudensky B (2004). Toll-like receptor 9 signaling mediates the anti-inflammatory effects of probiotics in murine experimental colitis. Gastroenterology.

[73] Liu X j, Yu R, Zou K f (2019). Probiotic mixture VSL#3 alleviates dextran sulfate sodium-induced colitis in mice by downregulating T follicular helper cells. Curr Med Sci.

[74] Veckman V, Miettinen M, Pirhonen J, Sirén J, Matikainen S, Julkunen I (2004). Streptococcus pyogenes and Lactobacillus rhamnosus differentially induce maturation and production of Th1-type cytokines and chemokines in human monocyte-derived dendritic cells. J Leukoc Biol.

[75] Spagnuolo R, Cosco C, Mancina RM, Ruggiero G, Garieri P, Cosco V (2017). Beta-glucan, inositol and digestive enzymes improve quality of life of patients with inflammatory bowel disease and irritable bowel syndrome. Eur Rev Med Pharmacol Sci.

[76] Welters CFM, Heineman E, Thunnissen FBJM, Van den Bogaard AEJM, Soeters PB, Baeten CGMI (2002). Effect of dietary inulin supplementation on inflammation of pouch mucosa in patients with an ileal pouch-anal anastomosis. Dis Colon Rectum.

[77] Vaarala O (2003). Immunological effects of probiotics with special reference to lactobacilli. Clin Exp Allergy.

[78] Drakes M, Blanchard T, Czinn S (2004). Bacterial probiotic modulation of dendritic cells. Infect Immun.

[79] Doron S, Snydman DR (2015). Risk and safety of probiotics. Clin Infect Dis.

[80] Eckersall PD, Bell R (2010). Acute phase proteins: biomarkers of infection and inflammation in veterinary medicine. Vet J.

[81] Rouissi A, Alfonso-Avila AR, Guay F, Boulianne M, Létourneau-Montminy MP. Effects of Bacillus subtilis, butyrate, mannan-oligosaccharide, and naked oat (ß-glucans) on growth performance, serum parameters, and gut health of broiler chickens. Poult Sci. 2021;100(12):101506. 10.1016/j.psj.2021.101506 PMC8571078 34731741

[82] Kan L, Guo F, Liu Y, Pham VH, Guo Y, Wang Z. Probiotics Bacillus licheniformis improves intestinal health of subclinical necrotic enteritis-challenged broilers. Front Microbiol. 2021;12:623739. 10.3389/fmicb.2021.623739 PMC8168541 34084155

[83] Pézsa NP, Kovács D, Rácz B, Farkas O. Effects of Bacillus licheniformis and Bacillus subtilis on gut barrier function, Proinflammatory response, ROS production and pathogen inhibition properties in IPEC-J2- Escherichia coli/ salmonella typhimurium co-culture. Microorganisms. 2022;10:10050936. 10.3390/microorganisms10050936 PMC9145911 35630380

[84] Skjolaas KA, Burkey TE, Dritz SS, Minton JE (2007). Effects of salmonella enterica serovar typhimurium, or serovar Choleraesuis, Lactobacillus reuteri and Bacillus licheniformis on chemokine and cytokine expression in the swine jejunal epithelial cell line, IPEC-J2. Vet Immunol Immunopathol.

[85] Zhou F-X, Chen L, Liu X-W, Ouyang C-H, Wu X-P, Wang X-H (2012). Lactobacillus crispatus M206119 exacerbates murine DSS-colitis by interfering with inflammatory responses. World J Gastroenterol.

[86] Feighery LM, Smith P, O’Mahony L, Fallon PG, Brayden DJ (2008). Effects of Lactobacillus salivarius 433118 on intestinal inflammation, immunity status and in vitro colon function in two mouse models of inflammatory bowel disease. Dig Dis Sci.

[87] Claes IJJ, Lebeer S, Shen C, Verhoeven TLA, Dilissen E, De Hertogh G (2010). Impact of lipoteichoic acid modification on the performance of the probiotic Lactobacillus rhamnosus GG in experimental colitis. Clin Exp Immunol.

[88] Mileti E, Matteoli G, Iliev ID, Rescigno M (2009). Comparison of the immunomodulatory properties of three probiotic strains of lactobacilli using complex culture systems: prediction for in vivo efficacy. PLoS One.

[89] Mohamadzadeh M, Pfeiler EA, Brown JB, Zadeh M, Gramarossa M, Managlia E (2011). Regulation of induced colonic inflammation by Lactobacillus acidophilus deficient in lipoteichoic acid. Proc Natl Acad Sci U S A.

[90] Mangalat N, Liu Y, Fatheree NY, Ferris MJ, van Arsdall MR, Chen Z, et al. Safety and tolerability of Lactobacillus reuteri DSM 17938 and effects on biomarkers in healthy adults: results from a randomized masked trial. PLoS One. 2012;7(9):0043910. 10.1371/journal.pone.0043910 PMC3435331 22970150

[91] Williams NT (2010). Probiotics. Am J Health Syst Pharm.

[92] Karpa KD (2007). Probiotics for Clostridium difficile diarrhea: putting it into perspective. Ann Pharmacother.

[93] Vermeire S, Joossens S, Peeters M, Monsuur F, Marien G, Bossuyt X (2001). Comparative study of ASCA (anti-Saccharomyces cerevisiae antibody) assays in inflammatory bowel disease. Gastroenterology.

[94] Underhill D, Braun J (2008). Current understanding of fungal microflora in inflammatory bowel disease pathogenesis. Inflamm Bowel Dis.

[95] Ge Y, Pan M, Zhang C, Wang C, Ma K, Yan G (2021). Paeonol alleviates dextran sodium sulfate induced colitis involving Candida albicans-associated dysbiosis. Med Mycol.

[96] Chiaro TR, Soto R, Stephens WZ, Kubinak JL, Petersen C, Gogokhia L, et al. A member of the gut mycobiota modulates host purine metabolism exacerbating colitis in mice. Sci Transl Med. 2017;9(380):9044. 10.1126/scitranslmed.aaf9044 PMC5994919 28275154

[97] Heinsbroek SEM, Williams DL, Welting O, Meijer SL, Gordon S, de Jonge WJ (2015). Orally delivered β-glucans aggravate dextran sulfate sodium (DSS)-induced intestinal inflammation. Nutr Res.

[98] Seifert S, Watzl B (2007). Inulin and Oligofructose: review of experimental data on immune modulation. J Nutr.

[99] Hutsko SL, Meizlisch K, Wick M, Lilburn MS (2016). Early intestinal development and mucin transcription in the young poult with probiotic and mannan oligosaccharide prebiotic supplementation. Poult Sci.

[100] Travis SPL, Stange EF, Lémann M, Øresland T, Bemelman WA, Chowers Y (2008). European evidence-based consensus on the management of ulcerative colitis: current management. J Crohn's Colitis.

[101] Garud S, Peppercorn MA (2009). Ulcerative colitis: current treatment strategies and future prospects. Ther Adv Gastroenterol.

[102] Mahida YR, Lamming CED, Gallagher A, Hawthorne AB, Hawkey CJ (1991). 5-Aminosalicylic acid is a potent inhibitor of interleukin 1β production in organ culture of colonic biopsy specimens from patients with inflammatory bowel disease. Gut.

[103] Shanahan F, Niederlehner A, Carramanzana N, Anton P. Sulfasalazine inhibits the binding of TNF alpha to its receptor. Immunopharmacology. 20(3):217–24. 10.1016/0162-3109(90)90037-f 1981213

[104] Kaur L, Gordon M, Baines PA, Iheozor-Ejiofor Z, Sinopoulou V, Akobeng AK (2020). Probiotics for induction of remission in ulcerative colitis. Cochrane Database Syst Rev.

[105] Shin MR, Kim KJ, Kim SH, Kim SJ, Il SB, An HJ, et al. Comparative evaluation between sulfasalazine alone and in combination with herbal medicine on DSS-induced ulcerative colitis mice. Biomed Res Int. 2017;2017:6742652. 10.1155/2017/6742652 PMC5606053 29018816

[106] Wadie W, Abdel-Aziz H, Zaki HF, Kelber O, Weiser D, Khayyal MT (2012). STW 5 is effective in dextran sulfate sodium-induced colitis in rats. Int J Color Dis.

[107] Molteni R (2011). EURL evaluation report on the analytical methods submitted in connection with the application for the authorisation of a feed additive according to regulation (EC) no 1831/2003.

[108] Dadashbeiki M, Sojoudi MR, Bouyeh M (2012). Effect of different levels of prebiotics TechnoMos on carcass characteristics of broiler chickens. J Basic Appl Sci Res.

[109] Ren J, Yue B, Wang H, Zhang B, Luo X, Yu Z, et al. Acacetin ameliorates experimental colitis in mice via inhibiting macrophage inflammatory response and regulating the composition of gut microbiota. Front Physiol. 2021;11:577237. 10.3389/fphys.2020.577237 PMC7848181 33536931

[110] Murthy SNS, Cooper HS, Shim H, Shah RS, Ibrahim SA, Sedergran DJ (1993). Treatment of dextran sulfate sodium-induced murine colitis by intracolonic cyclosporin. Dig Dis Sci.

[111] Grisham MB, Benoit JN, Neil GD (1990). Assessment of leukocyte involvement during ischemia and reperfusion of intestine. Methods Enzymol.

[112] Abdel-Rahman Mohamed A, Metwally MM, Khalil SR, Salem GA, Ali HA (2019). Moringa oleifera extract attenuates the CoCl2 induced hypoxia of rat’s brain: expression pattern of HIF-1α, NF-kB, MAO and EPO. Biomed Pharmacother.

[113] S. Kim Suvarna CL and JDB. Bancroft’s Theory and Practice of Histological Techniques. Bancroft’s Theory and Practice of Histological Techniques. 2019; 8th.

